# Elevated systemic interleukin-7 in patients with colorectal cancer and individuals at high risk of cancer: association with lymph node involvement and tumor location in the right colon

**DOI:** 10.1007/s00262-016-1933-3

**Published:** 2016-11-19

**Authors:** Malgorzata Krzystek-Korpacka, Marek Zawadzki, Katarzyna Neubauer, Iwona Bednarz-Misa, Sabina Górska, Jerzy Wiśniewski, Wojciech Witkiewicz, Andrzej Gamian

**Affiliations:** 1 Department of Medical Biochemistry, Wroclaw Medical University, ul. Chalubinskiego 10, 50-368 Wroclaw, Poland; 2 Department of Surgical Oncology, Regional Specialist Hospital, Wroclaw, Poland; 3 Department of Gastroenterology and Hepatology, Wroclaw Medical University, Wroclaw, Poland; 4 Laboratory of Medical Microbiology, Ludwik Hirszfeld Institute of Immunology and Experimental Therapy, Polish Academy of Sciences, Wroclaw, Poland; 5 Research and Development Centre at Regional Specialist Hospital, Wroclaw, Poland

**Keywords:** Interleukin-7, Colorectal cancer, Inflammatory bowel disease, Adenomas, Lymph node metastasis, Immunity

## Abstract

Interleukin (IL)-7 is a cytokine essential for protective immunity, and it is considered as a promising agent for cancer immunotherapy. Recent studies, however, appear to associate IL-7 with aggressiveness of solid tumors. The IL-7 has been less studied in colorectal cancer (CRC) and conditions associated with increased risk of CRC development. To explore IL-7 status in bowel diseases, it was measured immunofluorometrically in 431 individuals (110 with CRC) by using Luminex platform. A level of IL-7 in CRC patients was significantly higher than in controls, did not differ from those with adenomas, but was lower than in both active and inactive inflammatory bowel disease (IBD) cases. In CRC, IL-7 was higher in patients with lymph node and distant metastases and with tumors located in right colon. In adenomas, IL-7 elevation was associated exclusively with villous growth pattern, while in IBD, circulating IL-7 reflected clinical activity of Crohn’s disease and ulcerative colitis. Systemic TNFα, IL-10, and PDGF-BB were independent predictors of circulating IL-7. In summary, our study is the first to demonstrate IL-7 elevation in CRC in association with metastatic disease and tumor location. Both associations should be considered when designing IL-7-based immunotherapies for CRC. Further studies on IL-7 functionality in CRC are necessary.

## Introduction

Colorectal cancer (CRC) remains one of the commonest and most lethal cancers worldwide [1, 2]. CRC development is frequently preceded by formation of adenomas. Although benign, these lesions may develop into cancer through a sequence of genetic and epigenetic alterations. A prevalence of the adenomas in older population is relatively high [3, 4]. Inflammatory bowel disease (IBD) is another pathology closely associated with an increased risk of CRC development. Inflammatory character of the disease combined with its incurableness, chronicity, and relapsing-remitting nature eventually causes dysplasia and facilitates neoplastic transformation [5]. While survival rates for CRC patients with the resectable tumors are improving, the prognosis for patients with advanced CRC remains poor, rendering development of new therapeutic strategies a necessity. Immunotherapy, an approach based on boosting immune system to fight cancer, is a promising strategy that may improve outcomes for the CRC patients with metastatic disease or cancers resistant to chemo- and/or radiotherapy. It has also potential to prevent disease relapse, following radical tumor resection or eradication, facilitated by residual circulating cells, micrometastases or cancer stem cells [6].

Interleukin (IL)-7 is a pleiotropic cytokine, crucial for development and homeostasis of lymphocytes T, acting as their mitogen, growth and survival factor. IL-7 is ubiquitous and mainly tissue-derived cytokine [7–9]. Owing to its central role in innate and adaptive immunity, IL-7 has been listed as one of the “Top Agents with High Potential for Use in Treating Cancer” by the panel of experts at the National Cancer Institute Immunotherapy Agent Workshop in 2007 [10]. IL-7 may up-regulate tumor-directed immune responses in a number of ways, including, but not limited to, enhancement of the cellular (T_h_1) immune response or selective expansion of the tumor-redirected cytotoxic T lymphocytes [8, 9, 11, 12]. Unlike the other γ-chain cytokines tested as therapeutics (e.g. IL-2), application of IL-7 in cancer immunotherapy is particularly appealing, because it does not induce hyperinflammation [13, 14]. Since IL-7 plays a role in both predisposing to autoimmunity and in perpetuating autoimmune inflammation [7], targeting IL-7/IL7R signaling appears to be an attractive therapeutic option for preventing CRC [15].

However, there is an increasing number of reports showing IL-7 to be overexpressed by solid tumors [11, 16–19] and being elevated in sera of the cancer patients [20–24]. Although functional data are still scanty, available evidence seems to link IL-7 overexpression with tumor aggressiveness, metastasis, and unfavorable prognosis [11, 19].

In the light of growing interest in immunotherapy based on this cytokine and controversies associated with its expression by solid tumors, we aimed at exploring IL-7 status in CRC and conditions linked to an increased risk of its development, identifying factors contributing to this cytokine elevation.

## Materials and methods

### Study population

Systemic IL-7 was measured in serum samples from 431 individuals: 110 with CRC, 21 with adenomas, 171 with IBD (133 with active and 38 with inactive disease; 97 with Crohn’s disease (CD) and 74 with ulcerative colitis (UC)), and 129 controls. Enrolled CRC patients were admitted to the Department of Surgical Oncology, Regional Specialist Hospital in Wroclaw in years 2013–2015 for curative resection of histologically confirmed adenocarcinoma of colon or rectum. Patients aged <18 years, with poor overall physical status (ASA physical status classification system >3), requiring emergency surgery, with gross metastatic disease or locally advanced cancers not amenable to curative resection were excluded. Resected tumors were staged pathomorphologically according to UICC TNM 7th edition from 2010 and stage distribution is given in Table 1. Individuals with IBD and adenomas were inpatients of the Department of Gastroenterology and Hepatology of Wroclaw Medical University. Patients with indeterminate colitis or the co-existence of other severe systemic diseases, malignancies, liver diseases, or pregnancies were excluded. Crohn’s Disease Activity Index (CDAI) was applied for the assessment of CD activity and the Mayo Scoring System (MDAI) for UC. IBD patients, with few exceptions, were treated with 5′-aminosalicylate derivatives. Twelve patients with adenomas had multiple polyps, in nine the polyps were larger than 10 mm, and in eight the polyps had villous growth pattern. Control group consisted of healthy volunteers from hospital staff, outpatients of Research, Science, and Educational Center of Dementia Diseases, Scinawa, Poland, suffering from headaches or mild cognitive disorders (Alzheimer disease and other forms of dementia were excluded using neuroimaging and the following criteria: ICD-10, DSM IV, and NINCDS-ADRDA as revised in 2007), patients with non-malignant and non-inflammatory bowel diseases (irritable bowel syndrome, diverticulosis, hemorrhoids) and blood donors, whose sera were kindly provided by Regional Center of Blood Donation and Therapeutics in Wroclaw, Poland. Inclusion criteria for control group were: age >18 years, overall good health condition, and willingness to participate. Exclusion criteria were pregnancy, active inflammation, known severe systemic disease, dementia or depression.

**Table 1:** Circulating IL-7 and CRC advancement

	*N*	Mean IL-7 (95% CI)	*P*
Disease stage (UICC TNM7th)			0.027
0	5	5.95 (4–8.9)	
I	8	8.32 (5.9–11.9)	
II	41	10.59 (8.2–13.7)	
III	47	12.74 (10.7–15.1)	
IV	9	19.63 (7.2–80.7)	
Primary tumor (*T*)			0.162
Tis	5	5.95 (4–8.9)	
T1	1	7.45	
T2	11	10.73 (7.2–16)	
T3	62	11.19 (9.2–13.6)	
T4	31	14.22 (10.6–19.1)	
Lymph node involvement (*N*)			0.018
No (N0)	54	9.69 (7.9–11.9)	
Yes (N1/N2)	56 (33/23)	13.66 (11.2–16.7)	
Distant metastases (*M*)			0.030
M0	101	11.01 (9.6–12.6)	
M1	9	19.63 (72–53.6)	
Grade of differentiation (*G*)			0.693
G1	9	9.35 (4.4–19.8)	
G2	79	12.11 (10.1–14.5)	
G3	15	12.94 (10.5–16)	
G4	1	7.45	

Subjects distribution based on gender (females/males) in CRC, adenoma, active IBD, inactive IBD and control cohorts did not differ significantly (*p* = 0.673) and was as follows: 43/67, 11/10, 63/70; 16/22, and 57/72. Age distribution in CRC, adenoma, active IBD, inactive IBD, and in control cohorts was as follows: 65 years (95% CI 64–68), 61.5 years (56–77), 33 years (31–36), 40 years (29–44), and 50 years (42–56). Due to natural history of the diseases, median age in both IBD cohorts differed significantly from other groups (*p* < 0.05). However, IL-7 did not correlate with age in any evaluated cohort: *p* = 0.174 for CRC, *p* = 0.959 for adenomas, *p* = 0.156 for active IBD, *p* = 0.681 for inactive IBD, and *p* = 0.196 for controls. Nevertheless, control group was divided into subgroups based on age: <45 years (median age 34 years (33–38), *n* = 62) to match age distribution in IBD patients (*p* = 0.579) and ≥45 years (median age 64 years (62–65), *n* = 67) to match age distribution in CRC and adenoma patients (*p* = 0.295).

The study protocol was approved by the Medical Ethics Committees of Wroclaw Medical University and of Regional Specialist Hospital, and the study was conducted in accordance with the Helsinki Declaration of 1975, as revised in 1983, and an informed consent has been obtained from all patients.

### Analytical methods

Blood was drawn in a fasting state prior to any procedure by venipuncture, clotted for 30 min, and subsequently centrifuged (15 min, 720×*g*). Collected serum was aliquoted and kept frozen at −80° until examination. Samples were measured in duplicates or triplicates by means of flow cytometry-based method utilizing magnetic microspheres conjugated with monoclonal antibodies using the BioPlex 200 platform with HRF (Bio-Rad, USA), incorporating Luminex xMAP^®^ technology. Bio-Plex Pro™ Human Cytokine, Chemokine, and Growth Factor Magnetic Bead-Based Assays were used according to manufacturer’s instructions to measure the levels of IL-7 and IL-1β, IL-6, IL-8, IL-10, IL-12(p70), IFNγ, MCP-1, MIP-1α, MIP-1β, IP-10, FGF2, PDGF-BB, TNFα, and VEGF-A. Standard curves were drawn using 5-PL logistic regression, and the data were analyzed using BioPlex Manager 6.0 software.

### Statistical analysis

Chi-squared test was applied to assess the normality of data distribution. Homogeneity of variation was evaluated using Levene test. Data were log-transformed to obtain normality. Normally distributed data are presented as geometric means and analyzed using *t* test for independent samples with Welch correction if appropriate or one-way ANOVA with Bonferroni correction for multiple testing and Student–Newman–Keuls post hoc test. Non-normally distributed data are presented as medians and analyzed using Kruskal–Wallis *H* test. Both geometric means and medians are accompanied by 95% CI. Two-way ANOVA was employed to co-exam the effects of tumor location and regional metastases on IL-7. Correlation analysis was conducted using Spearman rank test (*ρ*) or Pearson test (*r*), depending on data character and distribution. Frequency analysis was conducted using Chi-square test. Enter and stepwise method of multivariate analysis was used to discern independent predictors of IL-7 and to determine partial correlation coefficients (net correlation with the effects of other variables removed). To limit the number of variables, we used two-step procedure. It allowed us to eliminate insignificant variables in a first step so the ratio of variables-to-cases in a final model was acceptable (1:16). The following criteria were used: *p* < 0.05 for entering and *p* > 0.1 for removing of variables. Coefficient of determination adjusted for the number of independent variables in the regression model (*R* ^2^-adjusted) represents the goodness of fit of the model. All calculated probabilities were two-tailed, and *p* values ≤0.05 were considered statistically significant. The statistical analysis was conducted using MedCalc Statistical Software version 16.2.0 (MedCalc Software bvba, Ostend, Belgium; https://www.medcalc.org; 2016).

## Results

### Circulating IL-7 in bowel diseases

**Fig. 1:**
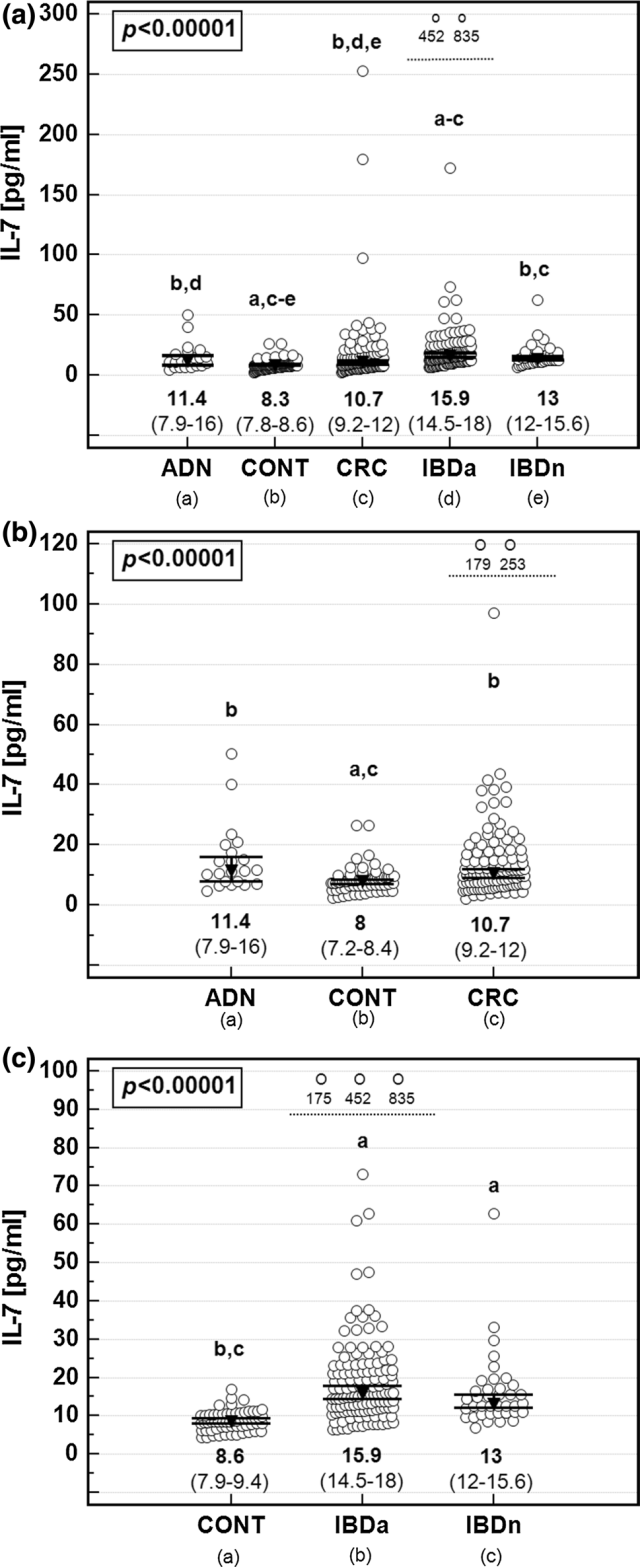
Systemic IL-7 in colorectal cancer and cancer high-risk conditions. **a** Analysis on whole study population; **b** Analysis limited to IBD patients and their age-matched controls; **c** Analysis limited to CRC and adenoma patients and their age-matched controls. ADN, adenomas; CONT, controls; CRC, colorectal cancer; IBDa, active inflammatory bowel disease; IBDn, inactive inflammatory bowel disease. Data presented as medians with 95% CI and analyzed using Kruskal–Wallis *H* test. *Lower script letters* indicate significant between-group differences. Outlying observations are presented together as *open circles* above *dashed line* and accompanied by IL-7 values

The concentration of IL-7 determined in sera from CRC patients was significantly higher than in controls, did not differ from patients with adenomas, and was significantly lower than in IBD, in both active and inactive cases of this disease (Fig. 1a).

Patients with adenomas had higher IL-7 than controls and lower than patients with active IBD, whose cytokine levels were significantly more elevated than in any other group except for inactive disease.

Owing to natural history of IBD and CRC with the diseases onset, respectively, early and late in life, the control group could not be age-matched to both CRC and IBD cohorts. Although systemic IL-7 did not correlate with age, we re-analyzed the data after dividing our controls into two subgroups: one including younger individuals (age <45 years) to be compared with IBD patients and the other consisting of older individuals (age ≥45 years) to be compared with adenoma and CRC patients. It allowed us to confirm significance of the elevation of IL-7 in CRC, adenoma, and IBD patients as compared to controls in age-matched analysis (Fig. 1b, c).

### Circulating IL-7 and CRC advancement

A relation between CRC advancement and serum concentration of IL-7 is summarized in Table 1. Cytokine levels increased along with the disease stage (*ρ* = 0.29, *p* = 0.002) with a significant difference between stages 0 and IV. Significant elevation in IL-7 concentration was associated also with lymph node involvement and presence of distant metastases. There was a weak positive correlation between IL-7 and T stage (*ρ* = 0.22, *p* = 0.021) and between IL-7 and a number of metastatic lymph nodes (*ρ* = 0.28, *p* = 0.013, *n* = 77).

### Circulating IL-7 and tumor location

**Fig. 2:**
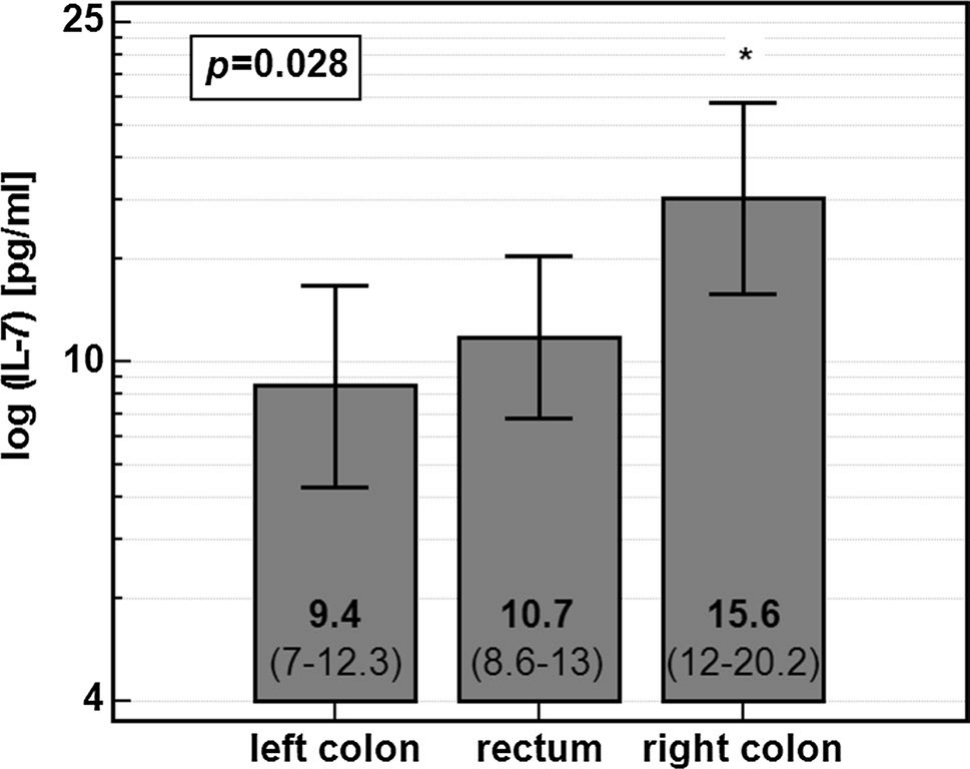
Circulating IL-7 and tumor location. Data presented as geometric means with 95% CI and analyzed using one-way ANOVA with Bonferroni corrections for multiple testing. *Asterisk* significantly different form other groups

Circulating IL-7 also differed with respect to tumor location. Its concentration was significantly higher in serum of the patients with tumors localized in right colon (Fig. 2).

There was no difference in stage distribution (*p* = 0.493), lymph node involvement (*p* = 0.811), or presence of distant metastases (*p* = 0.265) between right-sided CRC and other cancer locations. Moreover, when co-examined in two-way ANOVA, both lymph node involvement (*F* = 5.29, *p* = 0.023) and tumor location (*F* = 6.35, *p* = 0.013) were significantly and independently from each other associated with IL-7.

### Circulating IL-7 and CRC high-risk conditions

There were no differences in systemic IL-7 between CD and UC, regardless whether patients with active or non-active disease were compared. Cytokine levels positively correlated with the indices of clinical activity of both Crohn’s disease—CDAI (*ρ* = 0.3, *p* = 0.007) and ulcerative colitis—Mayo score (*ρ* = 0.3, *p* = 0.008).

In adenoma group, there was no difference in IL-7 with respect to number of polyps (one *vs*. multiple, *p* = 0.983) or their size (10 < vs. ≥ 10 mm, *p* = 0.993). However, IL-7 was significantly higher in patients with adenomas displaying a villous growth pattern (villous adenomas and tubulovillous adenomas) as compared to tubular adenomas: 20.6 pg/ml (95% CI 12.9–32.7) and 9.2 pg/ml (7.2–11.8), *p* = 0.001. Cytokine levels in patients with tubular adenomas did not differ significantly from controls (*p* = 0.182).

### Correlation pattern of IL-7 with other cytokines

We evaluated the association between IL-7 and other cytokines and growth factors selected from among pro-/anti-inflammatory and Th1/Th2 cytokines (IL-1β, IL-6, TNFα, IFNγ, IL12p(70), and IL-10), chemokines (MCP-1, MIP-1α, MIP-1β, and IL-8), and pro- and anti-angiogenic factors (IP10, FGF2, PDGF-BB, and VEGF-A). Circulating IL-7 was correlated with most of the evaluated cytokines and growth factors, except for MIP-1β and IP10. Particularly strong correlations were observed for TNFα and IFNγ (Table 2).

**Table 2:** IL-7 correlation pattern with inflammatory, Th1/Th2, chemotactic, and angiogenic cytokines

	Cytokine	Correlation coefficient, *p*
Inflammatory, Th1/Th2	IL-1β	0.52, *p* < 0.0001
	TNFα	0.65, *p* < 0.0001
	IL-6	0.40, *p* < 0.0001
	IL-12(p70)	0.42, *p* < 0.0001
	IFNγ	0.69, *p* < 0.0001
	IL-10	0.57, *p* < 0.0001
Chemotactic	MCP1	0.26, *p* = 0.007
	MIP1α	0.34, *p* < 0.001
	IL-8	0.21, *p* = 0.030
	MIP1β	0.17, *p* = 0.072
Angiogenic	FGF2	0.53, *p* < 0.0001
	PDGF-BB	0.54, *p* < 0.0001
	VEGF-A	0.20, *p* = 0.041
	IP10	0.13, *p* = 0.176

Statistically, the examined cytokines were intertwined and to discern their independent associations and determine partial correlation coefficients, we applied two-step multiple regression. First, we entered all cytokines significantly correlated with IL-7 in univariate analysis as independent variables. Then, cytokines with p > 0.1 were removed and remaining cytokines re-entered to analysis and stepwise method of model building was used. TNFα, IL-10, IL-1β, MCP-1, and PDGF-BB were entered in final model and TNFα, IL-10, and PDGF-BB remained as independent predictors of IL-7 explaining 64% of its variability (Table 3).

**Table 3:** Multiple regression analysis with IL-7 as dependent variable

	Partial correlation coefficient	statistics *t*	*p*
IL-10	0.494	5.78	<0.0001
PDGF-BB	0.411	4.63	0.002
TNFα	0.582	7.36	<0.0001
$R^{2}$_adjusted_	0.642		
*F* ratio, *p*	66.1, *p* < 0.0001		

## Discussion

In the context of cancer disease, interleukin (IL)-7 is viewed as a key therapeutic factor, while substantially less attention has been devoted to its possible association with the disease development. Occupying central position in innate and adaptive immunity, IL-7 with its repertoire of antibacterial, anti-fungal, anti-viral, and anti-tumor activities is considered as “a critical enhancer of protective immunity” [25]. At the same time, mitogenic activity of IL-7 toward lymphoid populations might result in lymphoproliferative diseases. Accordingly, the cytokine has been implicated in the development and progression of leukemias and lymphomas [11, 17]. IL-7 association with solid tumors is relatively new and unexplored aspect of the research. Nevertheless, there is a growing body of evidence linking IL-7/IL-7R signaling with tumor aggressiveness, metastatic disease, and unfavorable prognosis [11, 17, 19, 26]. There is paucity of data concerning IL-7 in CRC and conditions predisposing to the disease. IL-7 expression was found in tumor biopsies obtained from CRC patients [16], and it was reported by Crucitti et al. [21] to be significantly elevated in sera, while others linked IL-7 elevation to metastatic disease [20] and poorer survival [24]. Our findings confirm systemic elevation of IL-7 in CRC and expand the knowledge by placing it in the broad context of health and non-malignant bowel diseases associated with increased risk of CRC development.

Contrary to data presented by Henry et al. [27] but corroborating findings of Comstock et al. [28], we have observed that circulating IL-7 was elevated in patients with adenomas, as compared to controls. Similar increase in several other cytokines was reported elsewhere [28–31]. To resolve discrepancy between the reports, we investigated IL-7 in the context of adenomas, their number, size, and growth pattern. Detailed analysis revealed that IL-7 elevation coincided exclusively with villous growth pattern, while systemic IL-7 in patients with tubular adenomas was similar to controls. The dominant villous growth pattern is associated with large adenomas considered “advanced,” and they are more likely to progress into carcinoma [5]. The significance of IL-7 elevation in advanced adenomas needs further clarification, but it might be of interest to mention that in benign prostatic hyperplasia IL-7 has been shown to stimulate proliferation of the prostatic cells (reviewed in [17]).

Chronic inflammation-induced dysplasia is another risk factor for neoplastic transformation, and the odds of CRC development are six-times higher in IBD patients than in general population. Moreover, IBD patients are more likely to have multiple synchronous CRC and their mortality is higher than in a case of sporadic cancers [32]. Corroborating reports published by others [33–35], we found IL-7 to be elevated in IBD as compared to controls. IL-7 elevation was more pronounced than in CRC, regardless whether the disease was active or IBD patients were in remission. Still, IL-7 weakly corresponded with clinical activity of both Crohn’s disease and ulcerative colitis. IL-7 elevation observed in IBD patients is consistent with findings by others who reported higher percentage of activated and cycling CD4+ and CD8+ T cells and T cells expressing IFNγ as well as their increased resistance to apoptosis as compared to healthy individuals [15, 36]. The up-regulation of IL-7 signaling in IBD had a worse treatment-refractory disease course, probably due to enriched pool of antigen-specific memory T cells which, upon future re-encounter with antigen, would facilitate faster and more potent response from CD8 effector cells [37]. Intuitively, elevated IL-7 in IBD might confer at least some degree of protection against CRC development by promoting T cell mediated anti-tumor responses. However, T cell responsiveness to IL-7 has recently been demonstrated to be impeded by their exposure to inflammatory cytokines [38].

IL-7 is produced by stromal and epithelial cells as well as endothelial and immune cells. Its serum level is mainly regulated through the up-take by lymphocytes, thus elevating IL-7 in conditions associated with lymphopenia. The relevance of cytokine serum elevation found in cancer or the role of IL-7 expression and secretion by tumor cells remains elusive. Nonetheless, accumulated data seem to point at this cytokine involvement in metastasis [9, 11, 19]. Accordingly, our analysis of IL-7 association with clinicopathological features of CRC revealed that cytokine levels were more elevated in patients with lymph node involvement. Moreover, IL-7 levels were dependent on PDGF-BB and correlated with FGF2, VEGF-A, and IL-8, other potent angiogenic growth factors. Similar to Berghella’s et al. [20], we observed an increase in IL-7 in CRC patients with distant metastases despite a limited number of stage IV CRCs in current cohort. Our observations are consistent with lymphangiogenic role attributed to IL-7 [17, 39]. In lung cancer, IL-7 protein expression has correlated with increased lymphovascular density, lymph node metastases, advanced clinical stage, VEGF-D, and cyclin D expression [39] and has been identified as a potent marker for bone metastasis [19]. In fact, bone invasion has been required to trigger an intense cytokine production and its increase in sera [19]. Similar association with nodal involvement, tumor aggressiveness and worse prognosis was observed for IL-7 and expression of its receptor by Al-Rawi et al. [40] in breast cancer. Moreover, overexpression of IL-7R in lung adenocarcinomas was associated with tumor budding, which in turn has been associated with unfavorable outcome. The tumor-immune interactions at the invading edge of tumors have been advocated as a driving force for their aggressiveness [41]. Functionally, IL7 has been shown to induce expression of VEGF-D and cyclin D in A549 cells and promote proliferation and lymphangiogenesis of lung cancer xenograft tumors [39]. Moreover, exogenous IL-7 has been demonstrated to reduce the percentage of apoptotic non-small cell lung cancer cells by up-regulating the expression of anti-apoptotic Bcl-2 and down-regulating the expression of pro-apoptotic Bax and p53 [42].

An interesting observation reported in this study is a significant variation in systemic IL-7 levels with respect to tumor location. Although collectively referred to as colorectal cancer, the disease is heterogeneous and argued to encompass three entities, i.e., cancers of the right or left colon and rectal cancer [43, 44]. In general, the right colon cancers are considered to be more aggressive, poorly differentiated, and associated with worse prognosis than the left colon cancers [45]. To address the differences between these two colon cancers Glebov at el. [44] identified over 1300 genes that were differently expressed by right and left colon. However, the issue of possible differences being reflected on systemic level has not been explored. We observed IL-7 to be significantly more elevated in right than left or rectal cancers, and the association was not mediated by differences in stage or metastatic lymph node distribution. Taking into account advocated link between IL-7 signaling and tumor aggressiveness and hence worse prognosis [11, 17], more pronounced IL-7 elevation in right colon cancers might contribute to unfavorable outcomes associated with that tumor location. Interestingly, a total lymphocyte count was significantly lower in patients with right compare to left colon location of the tumors (data not shown). If systemic IL-7 reflects its intestinal expression, one of possible explanations of differences in IL-7 with respect to tumor location might be related to differences in bacterial load and composition in these two locations. Yoshioka et al. [46] reported that bacterial products such as flagellin might suppress IL-7 production by intestinal epithelial cells.

The rationale behind IL-7-based therapies is its capability to selectively expand subpopulations of T lymphocytes. IL-7 preferentially increases number of recent thymic emigrants, naïve, and central memory T cells [7] as well as tumor-redirected cytotoxic T lymphocytes [12]. In turn, expansion of the regulatory T cells is negligible, rendering their percentage in total lymphocyte population relatively decreased [7]. Similarly, by up-regulating the count of other subsets, IL-7 relatively reduces percentage of the senescent T cells [7]. Moreover, IL-7 protects T cells against tumor-induced senescence and thus abrogates their proangiogenic activity [47]. A number of researchers have recently reported successful application of IL-7. Wu et al. [48] used IL-7 to expand ex vivo Vδ1 T cells isolated from peripheral blood, which then was successfully used to restrain the tumor growth and improved survival of mice with xenografted human colon carcinoma. Zhao et al. [49] reported IL-7 insertion to autologous tumor vaccine modified with a virus that enhanced cytotoxicity and production of IFNγ of tumor-infiltrating CD8+ lymphocytes. CRC, like most other malignancies, is associated with unfavorable shift in immune responses toward Th2 and diminished total count of Th1 CD4+ cells, accompanied by reduced production of their prototypical cytokines IFNγ and IL-12 (reviewed in [50]). Here, we examined the associations between IL-7 and other cytokines and growth factors in CRC. IL-7 was tightly and positively correlated with Th1 cytokines IFNγ, TNFα and IL-12(p70) and Th2 cytokine IL-10, of which TNFα and IL-10 remained significantly correlated following adjustment to other associations. IL-7 dependence on TNFα is in agreement with stimulatory effect of TNFα on the expression of IL-7 [11]. We also observed IL-7 correlation with MCP-1 that might reflect cytokine effect on monocytes/macrophages. Zhang et al. [25] demonstrated that IL-7 induced macrophage activation and their homing into the gut, by inducing MCP-1 expression by intestinal epithelial cells.

In summary, our study is the first to demonstrate IL-7 elevation in CRC in association with lymph node involvement and tumor location. Both associations should be considered when designing IL-7-based immunotherapies. Further studies on IL-7 functionality in CRC are necessary.

## Abbreviations

ASA classification: American Society of Anesthesiologists classification of patients’ physical status
CD: Crohn’s disease
CDAI: Crohn’s disease activity index
CRC: Colorectal cancer
DSM IV: Diagnostic and statistical manual of mental disorders
FGF: Fibroblast growth factor
IBD: Inflammatory bowel disease
ICD-10: International statistical classification of diseases and related health problems
IP10: Interferon gamma-induced protein 10
MCP: Monocyte chemoattractant protein
MDAI: Mayo disease activity index
NINCDS-ADRDA: National Institute of Neurological and Communicative Disorders and Stroke-Alzheimer’s Disease and Related Disorders Association
PDGF: Platelet-derived growth factor
UC: Ulcerative colitis
UICC TNM classification: Union Internationale Contre le Cancer tumor-node-metastasis system for classification of malignant tumors
VEGF: Vascular endothelial growth factor

## References

[1] Siegel RL, Sahar L, Portier KM, Ward EM, Jemal A (2015). Cancer death rates in US congressional districts. CA Cancer J Clin.

[2] Chen W, Zheng R, Zeng H, Zhang S (2015). The updated incidences and mortalities of major cancers in China, 2011. Chin J Cancer.

[3] Armaghany T, Wilson JD, Chu Q, Mills G (2012). Genetic alterations in colorectal cancer. Gastrointest Cancer Res.

[4] Yamaji Y, Mitsushima T, Ikuma H, Watabe H, Okamoto M, Kawabe T (2004). Incidence and recurrence rates of colorectal adenomas estimated by annually repeated colonoscopies on asymptomatic Japanese. Gut.

[5] Fleming M, Ravula S, Tatishchev SF, Wang HL (2012). Colorectal carcinoma: pathologic aspects. J Gastrointest Oncol.

[6] Koido S, Ohkusa T, Homma S, Namiki Y, Takakura K, Saito K (2013). Immunotherapy for colorectal cancer. World J Gastroenterol.

[7] Lundström W, Fewkes N, Mackall CL (2012). IL-7 in human health and disease. Semin Immunol.

[8] Fry TJ, Mackall CL (2002). Interleukin-7: from bench to clinic. Blood.

[9] Capitini CM, Chisti AA, Mackall CL (2009). Modulating T cell homeostasis with IL-7: preclinical and clinical studies. J Intern Med.

[10] Sportès C, Babb RR, Krumlauf MC, Hakim FT, Steinberg SM, Chow CK (2010). Phase I study of recombinant human interleukin-7 administration in subjects with refractory malignancy. Clin Cancer Res.

[11] Gao J, Zhao L, Wan YY, Zhu B (2015). Mechanism of action of IL-7 and its potential applications and limitations in cancer immunotherapy. Int J Mol Sci.

[12] Perna SK, Pagliara D, Mahendravada A, Liu H, Brenner MK, Savoldo B (2014). Interleukin-7 mediates selective expansion of tumor-redirected cytotoxic T lymphocytes without enhancement of regulatory Tcell inhibition. Clin Cancer Res.

[13] Rosenberg SA, Sportès C, Ahmadzadeh M, Fry TJ, Ngo LT, Schwarz SL (2006). IL-7 administration to humans leads to expansion of CD8+ and CD4+ cells but a relative decrease of CD4+ T-regulatory cells. J Immunother.

[14] Sportès C, Hakim FT, Memon SA, Zhang H, Chua KS, Brown MR (2008). Administration of rhIL-7 in humans increases in vivo TCR repertoire diversity by preferential expansion of naive T cell subsets. J Exp Med.

[15] Caprioli F, Marafini I, Facciotti F, Pallone F, Monteleone G (2013). Targeting T-cells in chronic inflammatory bowel diseases. J Clin Cell Immunol.

[16] Maeurer MJ, Walter W, Martin D, Zitvogel L, Elder E, Storkus W (1997). Interleukin-7 (IL-7) in colorectal cancer: IL-7 is produced by tissues from colorectal cancer and promotes preferential expansion of tumour infiltrating lymphocytes. Scand J Immunol.

[17] Al-Rawi MA, Mansel RE, Jiang WG (2003). Interleukin-7 (IL-7) and IL-7 receptor (IL-7R) signalling complex in human solid tumours. Histol Histopathol.

[18] Mengus C, Le Magnen C, Trella E, Yousef K, Bubendorf L, Provenzano M (2011). Elevated levels of circulating IL-7 and IL-15 in patients with early stage prostate cancer. J Transl Med.

[19] Roato I, Caldo D, Godio L, D’Amico L, Giannoni P, Morello E (2010). Bone invading NSCLC cells produce IL-7: mice model and human histologic data. BMC Cancer.

[20] Berghella AM, Contasta I, Pellegrini P, Del Beato T, Adorno D (2002). Peripheral blood immunological parameters for use as markers of pre-invasive to invasive colorectal cancer. Cancer Biother Radiopharm.

[21] Crucitti A, Corbi M, Tomaiuolo PM, Fanali C, Mazzari A, Lucchetti D (2015). Laparoscopic surgery for colorectal cancer is not associated with an increase in the circulating levels of several inflammation-related factors. Cancer Biol Ther.

[22] Komura T, Sakai Y, Harada K, Kawaguchi K, Takabatake H, Kitagawa H (2015). Inflammatory features of pancreatic cancer highlighted by monocytes/macrophages and CD4+ T cells with clinical impact. Cancer Sci.

[23] Provatopoulou X, Georgiadou D, Sergentanis TN, Kalogera E, Spyridakis J, Gounaris A (2014). Interleukins as markers of inflammation in malignant and benign thyroid disease. Inflamm Res.

[24] Chen ZY, He WZ, Peng LX, Jia WH, Guo RP, Xia LP (2015). A prognostic classifier consisting of 17 circulating cytokines is a novel predictor of overall survival for metastatic colorectal cancer patients. Int J Cancer.

[25] Zhang W, Du JY, Yu Q, Jin JO (2015). Interleukin-7 produced by intestinal epithelial cells in response to *Citrobacter rodentium* infection plays a major role in innate immunity against this pathogen. Infect Immun.

[26] Ujiie H, Kadota K, Nitadori JI, Aerts JG, Woo KM, Sima CS (2015). The tumoral and stromal immune microenvironment in malignant pleural mesothelioma: a comprehensive analysis reveals prognostic immune markers. Oncoimmunology.

[27] Henry CJ, Sedjo RL, Rozhok A, Salstrom J, Ahnen D, Levin TR (2015). Lack of significant association between serum inflammatory cytokine profiles and the presence of colorectal adenoma. BMC Cancer.

[28] Comstock SS, Xu D, Hortos K, Kovan B, McCaskey S, Pathak DR (2016). Association of serum cytokines with colorectal polyp number and type in adult males. Eur J Cancer Prev.

[29] Kim S, Keku TO, Martin C, Galanko J, Woosley JT, Schroeder JC (2008). Circulating levels of inflammatory cytokines and risk of colorectal adenomas. Cancer Res.

[30] Krzystek-Korpacka M, Diakowska D, Kapturkiewicz B, Bebenek M, Gamian A (2013). Profiles of circulating inflammatory cytokines in colorectal cancer (CRC), high cancer risk conditions, and health are distinct. Possible implications for CRC screening and surveillance. Cancer Lett.

[31] Krzystek-Korpacka M, Diakowska D, Neubauer K, Gamian A (2013). Circulating midkine in malignant and non-malignant colorectal diseases. Cytokine.

[32] Mattar MC, Lough D, Pishvaian MJ, Charabaty A (2011). Current management of inflammatory bowel disease and colorectal cancer. Gastrointest Cancer Res.

[33] Watanabe M, Watanabe N, Iwao Y, Ogata H, Kanai T, Ueno Y (1997). The serum factor from patients with ulcerative colitis that induces T-cell proliferation in the mouse thymus is interleukin-7. J Clin Immunol.

[34] Kader HA, Tchernev VT, Satyaraj E, Lejnine S, Kotler G, Kingsmore SF (2005). Protein microarray analysis of disease activity in pediatric inflammatory bowel disease demonstrates elevated serum PLGF, IL-7, TGF-beta1, and IL-12p40 levels in Crohn’s disease and ulcerative colitis patients in remission versus active disease. Am J Gastroenterol.

[35] Korolkova OY, Myers JN, Pellom ST, Wang L, M’Koma AE (2015). Characterization of serum cytokine profile in predominantly colonic inflammatory bowel disease to delineate ulcerative and Crohn’s colitides. Clin Med Insights Gastroenterol.

[36] Funderburg NT, Stubble Park SR, Sung HC, Hardy G, Clagett B, Ignatz-Hoover J (2013). Circulating CD4+ and CD8+T-cells are activated in inflammatory bowel disease and are associated with plasma markers of inflammation. Immunology.

[37] Lee JC, Lyons PA, McKinney EF, Sowerby JM, Carr EJ, Bredin F (2011). Gene expression profiling of CD8+ T-cells predicts prognosis in patients with Crohn disease and ulcerative colitis. J Clin Investig.

[38] Shive CL, Mudd JC, Funderburg NT, Sieg SF, Kyi B, Bazdar DA (2014). Inflammatory cytokines drive CD4+ T-cell cycling and impaired responsiveness to interleukin 7: implications for immune failure in HIV disease. J Infect Dis.

[39] Ming J, Zhang QF, Jiang YD, Jiang GC, Qiu XS (2015). Anti-lymphangiogenesis effects of a specific antiinterleukin 7 receptor antibody in lung cancer model in vivo. Mol Carcinog.

[40] Al-Rawi MA, Rmali K, Watkins G, Mansel RE, Jiang WG (2004). Aberrant expression of interleukin-7 (IL-7) and its signaling complex in human breast cancer. Eur J Cancer.

[41] Kadota K, Yeh YC, Villena-Vargas J, Cherkassky L, Drill EN, Sima CS (2015). Tumor budding correlates with the protumor immune microenvironment and is an independent prognostic factor for recurrence of stage I lung adenocarcinoma. Chest.

[42] Liu ZH, Wang MH, Ren HJ, Qu W, Sun LM, Zhang QF (2014). Interleukin 7 signaling prevents apoptosis by regulating bcl-2 and bax via the p53 pathway in human non-small cell lung cancer cells. Int J Clin Exp Pathol.

[43] Li F, Lai M (2009). Colorectal cancer, one entity or three. J Zhejiang Univ Sci B.

[44] Glebov OK, Rodriguez LM, Nakahara K, Jenkins J, Cliatt J, Humbyrd CJ (2003). Distinguishing right from left colon by the pattern of gene expression. Cancer Epidemiol Biomarkers Prev.

[45] Derwinger K, Gustawsson B (2011). Variations in demography and prognosis by colon cancer location. Anticancer Res.

[46] Yoshioka A, Okamoto R, Oshima S, Akiyama J, Tsuchiya K, Nakamura T (2008). Flagellin stimulation suppresses IL-7 secretion of intestinal epithelial cells. Cytokine.

[47] Ramello MC, Boari JT, Canale FP, Mena HA, Negrotto S, Gastman B (2014). Tumor-induced senescent T cells promote the secretion of pro-inflammatory cytokines and angiogenic factors by human monocytes/macrophages through a mechanism that involves Tim-3 and CD40L. Cell Death Dis.

[48] Wu D, Wu P, Wu X, Ye J, Wang Z, Zhao S (2015). Ex vivo expanded human circulating Vδ1 γδT cells exhibit favorable therapeutic potential for colon cancer. Oncoimmunology.

[49] Zhao L, Mei Y, Sun Q, Guo L, Wu Y, Yu X (2014). Autologous tumor vaccine modified with recombinant new castle disease virus expressing IL-7 promotes antitumor immune response. J Immunol.

[50] Evans C, Dalgleish AG, Kumar D (2006). Immune suppression and colorectal cancer. Aliment Pharmacol Ther.

